# Alternative Splicing in Tumorigenesis and Cancer Therapy

**DOI:** 10.3390/biom15060789

**Published:** 2025-05-29

**Authors:** Huiping Chen, Jingqun Tang, Juanjuan Xiang

**Affiliations:** 1 Hunan Key Laboratory of Early Diagnosis and Precise Treatment of Lung Cancer, The Second Xiangya Hospital, Central South University, Changsha 410013, China; 236511071@csu.edu.cn; 2 Cancer Research Institute, School of Basic Medical Science, Central South University, Changsha 410078, China; 3 NHC Key Laboratory of Carcinogenesis and the Key Laboratory of Carcinogenesis and Cancer Invasion of the Chinese Ministry of Education, Xiangya Hospital, Central South University, Changsha 410078, China; 4 Department of Thoracic Surgery, The Second Xiangya Hospital, Central South University, Changsha 410013, China

**Keywords:** alternative splicing, cancer, immunotherapy, neoantigens, innovative technologies

## Abstract

Alternative splicing (AS) is a pivotal post-transcriptional mechanism that expands the functional diversity of the proteome by enabling a single gene to generate multiple mRNA and protein isoforms. This process, which involves the differential inclusion or exclusion of exons and introns, is tightly regulated by splicing factors (SFs), such as serine/arginine-rich proteins (SRs), heterogeneous nuclear ribonucleoproteins (hnRNPs), and RNA-binding motif (RBM) proteins. These factors recognize specific sequences, including 5′ and 3′ splice sites and branch points, to ensure precise splicing. While AS is essential for normal cellular function, its dysregulation is increasingly implicated in cancer pathogenesis. Aberrant splicing can lead to the production of oncogenic isoforms that promote tumorigenesis, metastasis, and resistance to therapy. Furthermore, such abnormalities can cause the loss of tumor-suppressing activity, thereby contributing to cancer development. Importantly, abnormal AS events can generate neoantigens, which are presented on tumor cell surfaces via major histocompatibility complex (MHC) molecules, suggesting novel targets for cancer immunotherapy. Additionally, splice-switching oligonucleotides (SSOs) have shown promise as therapeutic agents because they modulate splicing patterns to restore normal gene function or induce tumor-suppressive isoforms. This review explores the mechanisms of AS dysregulation in cancer, its role in tumor progression, and its potential as a therapeutic target. We also discuss innovative technologies, such as high-throughput sequencing and computational approaches, that are revolutionizing the study of AS in cancer. Finally, we address the challenges and future prospects of targeting AS for personalized cancer therapies, emphasizing its potential in precision medicine.

## 1. Introduction

AS is a cellular process that enables a single gene to generate multiple mRNA and protein isoforms by joining exons in different combinations. AS is a crucial mechanism for gene product diversity, leading to diverse protein isoforms with unique functions [1,2]. This approach provides significant evolutionary advantages by expanding the functional repertoire of genes. There are five primary modes of AS: exon skipping, alternative 5′ splice sites, alternative 3′ splice sites, intron retention and mutually exclusive exons [3,4,5,6,7,8]. AS events are regulated by a group of SFs, including SRs, hnRNPs, and RBM proteins [9,10,11,12]. These SFs recognize and bind to specific sequences, such as the 5′ splice site, 3′ splice site, and branch point, as well as exonic and intronic splicing enhancers (ESEs/ISEs) and silencers (ESSs/ISSs), to ensure precise and efficient splicing [13,14].

Abnormal AS can serve as a biomarker and therapeutic target for cancer. Dysregulation of AS can lead to the production of oncogenic splice variants that promote tumor growth, metastasis, and therapy resistance. Additionally, such abnormalities can cause the loss of tumor-suppressing activity, thereby contributing to cancer development. The identification of neoantigens resulting from abnormal AS events in cancer cells provides new therapeutic targets [1,15,16]. These neoantigens can be presented by MHC molecules on the surface of tumor cells, triggering immune responses and offering new avenues for immunotherapy. Additionally, splice-switching oligonucleotides (SSOs) have emerged as powerful tools to correct aberrant splicing or induce the expression of therapeutic splice variants [17]. By targeting specific splicing variants, SSOs can restore normal gene function or increase the production of tumor-suppressive isoforms. In this review, we explore the role of AS in cancer biology and therapy, focusing on the mechanisms underlying aberrant splicing, its impact on tumor progression, and its potential as a therapeutic target. We also discuss innovative technologies, such as high-throughput sequencing and computational tools, that are advancing our understanding of AS in cancer. Finally, we highlight the challenges and future directions in targeting AS for cancer treatment, emphasizing the potential of personalized therapies on the basis of splicing events.

## 2. Normal and Abnormal Alternative Splicing

AS is a normal phenomenon in eukaryotes. Approximately 95% of multiexonic genes are alternatively spliced to produce alternative transcript products from the same gene. However, the functional significance of these isoforms remains a subject of debate. While some scientists argue that most splice variants are due to splicing errors and lack functional relevance, others believe that some isoforms play critical roles in cellular processes. Studies indicate that fewer than 10% of genes produce functionally distinct splice isoforms (FDSIs) [18], with more than 90% of detected splice variants absent at the protein level [19]. Unproductive AS generally introduces premature termination codons and undergoes rapid nonsense-mediated decay (NMD) [20]. FDSIs are defined as those in which at least two variants are necessary for the gene’s normal function. Phenotypic analyses, including RNAi knockdown and isoform-specific rescue experiments, have identified only a limited number of genes with functionally distinct isoforms. In cases where the depletion of one splice isoform of a gene causes a phenotype, the depletion of the remaining splice isoforms of the same gene does not generate the same phenotype [18]. Notably, some studies have shown that different splice isoforms can rescue the same phenotype, suggesting functional redundancy. Fewer than 10% of human protein isoforms in UniProt have experimentally validated functional annotations [18]. Most alternatively spliced isoforms are expressed at low levels and lack cross-species conservation, suggesting that the majority of isoforms are nonfunctional transcripts resulting from mis-splicing [20]. There are several examples of normal AS, where specific isoforms play essential roles in regulating cellular functions and maintaining physiological homeostasis [21,22,23,24,25,26,27,28,29,30]. A notable example is the TP53, a crucial tumor suppressor gene, which produces multiple isoforms through alternative splicing, including full-length p53α, as well as Δ40p53, p53β, and p53γ. These isoforms have distinct roles in regulating genomic stability, apoptosis, tissue repair, and development. These isoforms are vital for both normal physiological processes and pathological states, such as cancer, by maintaining cellular and tissue balance.

## 3. Abnormal Alternative Splicing and Cancer

While normal AS plays a role in physiological processes, aberrant splicing is a hallmark of many diseases, including cancer and genetic disorders [31,32,33]. In cancers, splicing abnormalities are particularly prevalent, with tumors exhibiting up to 30% more AS events than normal samples [34]. These aberrant splicing events can generate cancer-specific isoforms that confer growth benefits and promote tumorigenesis [35]. Chemoresistant cancer cells often show a reduction in AS events, which may increase their survival under treatment [36]. Mutations that disrupt splicing regulation or cause reading frameshifts can lead to the production of dysfunctional proteins. Aberrant AS in cancer mainly arises from mutations in cis-acting regulatory elements, trans-acting SFs, and small nuclear RNAs (snRNAs), as well as the dysregulated expression of SFs [37].

### 3.1. Cis-Acting Splicing Mutations

A splice-site mutation refers to a genetic alteration in the DNA sequence that occurs at the junction of an exon and an intron (splice site). This change can disrupt normal RNA splicing, resulting in aberrant splicing patterns and an altered protein-coding sequence. Cis splicing mutations, which account for 15–60% of human disease-causing mutations, often disrupt splice site signals or splicing enhancer/silencer elements within pre-mRNAs. This disruption can lead to the production of aberrant mRNA and protein products [38]. Recurrent somatic point mutations near splicing sites can facilitate or inhibit specific splicing changes, resulting in erroneous splicing of cancer-associated genes and the generation of new splice isoforms [39,40,41]. Many driver mutations are thought to promote cancer through aberrant splicing. A notable example is mutations in the splice junctions of the MET gene, which lead to the skipping of exon 14. This AS results in a truncated MET protein that exhibits oncogenic activity and has clinical significance in lung adenocarcinoma (LUAD) [42]. This could serve as a druggable target, and detailed content will be discussed in the section “Targeting Novel Splice Variants”. A statistical framework for the full landscape of splice-altering variants (SAVs) was applied to whole exome and transcriptome sequencing data from 8976 cancer samples, systematically identifying 14,438 SAVs, approximately 50% of which disrupt or create splice sites. This study revealed the genomic features of SAVs, potential mutational processes, and their impact on cancer driver genes, including *TP53*, *PIK3R1*, *GATA3*, and *CDKN2A*. For example, TP53 splice site mutations often result in exon skipping or intron retention, resulting in the production of a nonfunctional p53 protein that fails to suppress tumor growth. Similarly, PIK3R1 mutations can dysregulate the PI3K signaling pathway, promoting cell survival and proliferation, while CDKN2A mutations can lead to loss of p16 function, disrupting cell cycle control [43].

### 3.2. Chromatin State

Splicing often occurs cotranscriptionally, suggesting that the chromatin state affects AS [44]. Nucleosomes localize to exons, with histone modifications such as H3K36me3 and H3K9me3, along with DNA methylation, forming a chromatin landscape for exon recognition and splicing. RNA-guided mechanisms, via small noncoding RNAs, influence splicing by recruiting histone-modifying enzymes. The kinetic coupling model shows that the transcription elongation rate impacts splicing. Slower rates facilitate weak splice site recognition and promote alternative exon inclusion. Histone methylation and acetylation modulate RNA polymerase II movement, affecting elongation and splicing [45]. For example, H3K9ac is related to fast elongation, whereas H3K36me3 is linked to slower elongation and more exon inclusion [46]. In splicing regulation, H3K36me3, which is abundant in exons, recruits SFs such as PTB and MRG15 to promote exon inclusion [47], whereas H3K9me3, a heterochromatin marker, is associated with exon skipping. DNA methylation near splice sites, regulated by factors like MeCP2, also influences splicing [48]. In colorectal cancer (CRC), the presence of H3K9me3 suppresses the transcription elongation rate of RNA polymerase II, which induces increased skipping of the CD44 exon, contributing to a partial epithelial-to-mesenchymal transition (EMT). This alteration in splicing promotes the invasion and metastasis of cancer cells in CRC, highlighting the role of chromatin modification in cancer progression [49].

### 3.3. RNA Structure

The structures of pre-mRNAs can influence AS by altering the function of splicing regulatory elements and proteins [50]. Secondary structures, such as hairpins, can either mask or expose splice sites, affecting the binding of SFs and small nuclear ribonucleoproteins (snRNPs). Stable structures near splice sites can prevent spliceosome recognition, causing exon skipping or intron retention. Conversely, the unfolding of these structures can enhance splice site recognition and promote exon inclusion, often facilitated by RNA-binding proteins (RBPs). Epigenetic RNA modifications, particularly N6-methyladenosine (m6A), play an important role in modulating AS. m6A, a prevalent RNA modifications, is found abundantly in both exons and introns. m6A-modified exons have a higher likelihood of being retained in mature mRNAs [51]. For instance, in oesophageal carcinoma [52], a negative correlation has been reported between m6A patterns and AS features in individual patients. The “m6A writer” complex, with METTL3 as the core enzyme, adds m6A to target RNAs, influencing AS in many genes, including MDM4, MDM2, FAS, BAX, and VEGFA [51,53]. In turn, AS can affect m6A deposition or recognition on mRNAs [51]. This dynamic relationship between AS and m6A deposition suggests that alterations in RNA structure and modifications can have profound implications for cancer biology.

### 3.4. Trans-Acting Splicing Mutations

Trans-acting splicing regulators such as SFs and RBPs are crucial in cancer splicing [38,54]. SF mutations are common and can cause mistakes in tumor suppressor gene mRNA splicing. For example, in pancreatic ductal adenocarcinoma (PDAC) [55] and lymphoid leukemia [56], SF3B1 (a U2 snRNP core) missense mutations lead to 3′ splice site recognition errors and abnormal splicing of tumor suppressor genes [57,58]. In sonic hedgehog medulloblastoma, U1 snRNP mutations disrupt mRNA splicing, inactivating genes such as PTCH1 and activating GLI2 [59,60,61,62,63]. An analysis of 32 cancer types revealed many splicing events linked to SF mutations, such as those in SF3B1 and U2AF1 [34]. The RBM family, which is important for alternative splicing regulation, is also key in cancers. RBM dysregulation from mutations or altered expression can produce abnormal splicing isoforms that drive tumor growth. In non-small cell lung cancer (NSCLC) [7], RBM4 exon 3 skipping changes RBM4-FL to RBM4-S. RBM4-FL inhibits the SRSF1-mTORC1 pathway, but RBM4-S does not, making the pathway hyperactive and promoting NSCLC cell growth [2]. In colorectal cancer (CRC), the regulatory role of RBM39 in splicing changes CDK5RAP2 from its long isoform to its short isoform, promoting CRC development [8,15].

### 3.5. Abnormal AS and NMD Regulation

NMD is a key cellular surveillance mechanism that degrades aberrant mRNAs containing premature termination codons (PTCs), which often arise from unproductive AS events [64]. Abnormal AS products can evade NMD through various mechanisms, such as altering splice sites, modulating mRNA stability, changing RNA secondary structures, or influencing interactions with RBPs [65]. These evasion strategies can lead to the accumulation of dysfunctional or truncated proteins, contributing to disease pathogenesis, including cancer pathogenesis.

Various cellular stresses, including amino acid deprivation, hypoxia, nutrient deprivation, infection, reactive oxygen species (ROS), and double-stranded RNA, can inhibit NMD [66]. The stress-induced inhibition of NMD is primarily mediated by the phosphorylation of eukaryotic initiation factor 2α (eIF2α), a key regulatory step in the integrated stress response (ISR) [67]. Phosphorylated eIF2α reduces global translation while selectively enhancing the expression of stress-responsive genes, such as ATF4, which plays a crucial role in cellular adaptation to stress. By dynamically regulating NMD, tumors can adapt to the tumor microenvironment (TME), such as hypoxia and nutrient deprivation, thereby promoting survival, proliferation, and metastasis. The inhibition of NMD in the TME may dynamically regulate the expression of key genes involved in tumorigenesis and cellular stress responses.

Under certain circumstances, activated NMD may paradoxically promote cancer progression. In myelodysplastic syndromes (MDS) [68], mutations in the SRSF2 induce aberrant recognition of specific exons, generating splice isoforms containing PTCs. These defective transcripts are targeted by NMD, leading to significant downregulation of critical genes EZH2 and INTS3. This NMD-driven depletion synergizes with RAS pathway activation, collectively contributing to the malignant transformation of MDS into acute leukemia.

### 3.6. Abberant Splicing in Breast Cancer

Aberrant splicing of genomic loci, including those encoding estrogen receptors (ERs), HER2/neu, Cyclin D1, BRCA1, BARD1, Tenscin-C, and CD44, has been implicated in breast carcinogenesis [69]. The ER gene produces multiple splice variants, including ERα66, ERα36 and ERα46. Among these, ERα36, a splice variant from the ESR1 locus, plays an important role in governing nongenomic membrane signaling pathways triggered by estrogen and confers 4-hydroxytamoxifen resistance in breast cancer therapy [69]. High levels of ERα36 have been associated with reduced benefits from endocrine therapy, indicating its role in mediating tamoxifen resistance. The balance between ERα66 and ERα36 impacts the antitumorigenic effects of vitamin D3 and its metabolites.

HER2, a known driver of breast cancer, undergoes AS resulting in variants like HER2 lacking exon 20 (Δ16HER2) with increased transforming ability compared to the wild-type HER2. This alteration can mimic phenotypes observed in endocrine therapy-resistant breast cancer cases.

The CD44 gene produces multiple splice variants, including the standard isoform (CD44s) and the variable isoform (CD44v), each involved in essential roles in breast cancer development. The CD44v subpopulation in 4T1 breast cancer cells exhibits increased metastatic potential through the expansion of stem-like cancer cells [70]. However, some studies indicate an association of CD44v with the luminal A subtype of breast cancer, which generally has a better prognosis [69], while the CD44 variant v3–10 has shown superior effectiveness compared to the standard isoform in slowing tumor growth and metastasis [71]. A chimeric monoclonal antibody that recognizes the CD44v6 isoform has demonstrated potential for radioimmunotherapy [72]. In clinical trials, this antibody labeled with 186Re could detect up to 66% of breast cancer lesions [73], underscoring its diagnostic and therapeutic potential.

### 3.7. Abberant Splicing in Colorectal Cancer

The KRAS gene is one of the most commonly mutated genes in human cancers, particularly in CRC, LUAD, and PDAC [74]. Despite its significant role in tumorigenesis, therapeutic strategies specifically targeting mutated KRAS are currently lacking. The functions and cell type-specific expression of the two known proteins produced by the KRAS locus have been described in normal tissues and during tumorigenesis. The human KRAS locus produces two distinct protein isoforms, KRAS4A and KRAS4B, through AS [75]. The less common KRAS4A isoform is prominently present in cancer stem-like cells and is responsive to hypoxic conditions, while the prevalent KRAS4B isoform is upregulated in response to endoplasmic reticulum (ER) stress [76]. Notably, the deletion of KRAS4A has been shown to suppress cancer stem cells. The abundance of the minor KRAS4A isoform in human tumors may serve as a biomarker for sensitivity to specific cancer treatment [76]. The primary developmental functions of KRAS are mediated through the KRAS4B isoform, whereas KRAS4A plays a critical role in cancer progression, possibly through effects on a minor population of stem cells [76]. Targeting RBM39/DCAF15, a key regulator of KRAS mRNA splicing, has demonstrated promising potential in inhibiting cancer stem cells [76].

### 3.8. Abberant Splicing in Lung Cancer

Aberrant splicing of key genes, including EGFR, KRAS, TP53, BCL2 and MET, plays a significant role in the pathogenesis of lung cancer, impacting tumor behavior and treatment responses.

The T790M mutation in EGFR, known as the “gatekeeper” mutation, confers resistance to first- and second-generation EGFR tyrosine kinase inhibitors (TKIs) by impeding drug binding to the ATP cleft [77]. Additionally, AS variants of EGFR have been identified in tumor cells and are being explored as potential targets for T-cell receptor (TCR)-based therapies [78]. HER2 amplification has been identified as a driver of first- and second-generation EGFR TKI resistance in tumors lacking T790M EGFR mutations [79]. The exon 16-skipping splice variant of HER2, known as HER2D16, has been recognized as a mediator of osimertinib resistance in patients with metastatic EGFR-mutant NSCLC.

*TP53*, a frequently mutated tumor suppressor gene in lung cancer, undergoes AS resulting in various isoforms. These isoforms include dominant-negative variants that exhibit contrasting functions to p53WT, as well as isoforms with distinct functions and regulatory roles in cellular processes. The presence of specific TP53 isoforms can influence tumor behavior, the response to therapy, and patient prognosis [80]. The activation of full-length p53 typically results in cellular apoptosis, whereas the induction of the AS isoform, the beta isoform of p53 (p53β), results in cellular senescence [81]. NSCLC cells expressing the Δ40p53 and Δ133p53 isoforms and treated with cisplatin exhibits enhanced apoptosis [82], and the Δ133p53 isoform can be a novel transcriptional enhancer of T-cell effector function to improve T-cell-based cancer immunotherapy [83].

*Bcl-X*, a critical apoptotic gene of BCL2 function, produces two antagonistic isoform splice variants, Bcl-Xl and Bcl-Xs, through AS. The expression of these variants can contribute to resistance against chemotherapeutic agents, highlighting the importance of splicing in therapeutic responses. Increased expression of Bcl-xL after irradiation promotes the malignant actions of lung cancer cells [84].

MET exon 14 skipping mutations occur in approximately 3–5% of NSCLCs, resulting in truncated MET receptor lacking the juxtamembrane regulatory domain, leading to abnormal MET signaling and oncogenesis. MET exon 14 skipping mutations represent a targetable alteration [85].

### 3.9. Other Malignancies

The BCL-2 gene, a critical regulator of apoptosis, initially identified for its role in B-cell lymphoma, produces two major isoforms through AS: BCL-2α and BCL-2β. BCL-2α is well known for its antiapoptotic properties, while the less characterized BCL-2β lacks exon 3, resulting in the absence of a transmembrane-anchoring domain [86]. Despite the difference, BCL-2β retains the same BH domains and general structure found in BCL-2α. BCL-2β features a unique 9-amino acid stretch at its C-terminal domain, distinguishing it from its α counterpart.

In acute myeloid leukemia (AML), mutations in the FLT3 gene often lead to aberrant splicing, generating constitutively active forms of the FLT3 receptor tyrosine kinase. These splice variants drive the uncontrolled proliferation and survival of leukemic cells. Particularly, internal tandem duplications (FLT3-ITDs) of FLT3 mutations are associated with poor prognosis and resistance to conventional therapies. Targeting FLT3 splice variants or their downstream signaling pathways emerges as a promising therapeutic strategy in AML [87].

In glioblastoma (GBM), the EGFRvIII mutation is a well-known driver of tumorigenesis. This mutation results in the deletion of exons 2–7, producing a constitutively active EGFR variant that promotes tumor growth and survival [88]. Recent studies have characterized the genome-wide AS induced by EGFRvIII in GBM, revealing its impact on tumor cell metabolism and transformation. EGFRvIII upregulates hnRNP A1 in SFs, which plays a key role in AS. hnRNP A1 promotes splicing of the Max transcript, generating a truncated isoform called Delta Max. Delta Max, unlike the full-length Max protein, enhances Myc-dependent transformation and metabolic reprogramming in GBM cells. Specifically, Delta Max rescues Myc-dependent glycolytic gene expression even after the loss of EGFRvIII, highlighting its role in sustaining tumor cell metabolism [89]. Table 1 summarizes the key abnormal AS of genes across the aforementioned cancer types.

### 3.10. AS and Cancer Immunotherapy

#### 3.10.1. AS and Immune Activation

Immune therapy, including immune checkpoint blockade (ICB), adoptive cell therapy (ACT), and oncolytic virus therapy, has revolutionized the treatment landscape of various malignant tumors. T cell-based immunotherapies show promise in treating cancer by targeting cancer-specific antigens, but are limited in tumors with low mutations and high heterogeneity. Identification of novel tumor-wide neoantigens from RNA splicing erros, such as GNAS and RPL22, enhances T cell therapy by recognizing and eradicating cancer cells across various tumor types [106]. Neoantigens, recognized as nonself antigens newly formed by tumor cells due to various tumor-specific alterations such as genomic mutations, dysregulated RNA splicing, disordered post-translational modifications, and integrated viral ORFs, hold significant therapeutic potential [37]. Tumor cells undergo numerous AS events, resulting in the production of unique splicing isoforms absent in normal cells [107,108]. These splice isoforms can be translated into protein variants containing new epitopes [109,110,111,112], which act as neoantigens recognized by MHC molecules, particularly MHC I, and presented to CD8^+^ T cells, triggering an immune response [113,114,115]. The tumor mutational burden (TMB), representing the number of somatic nonsynonymous mutations per megabase in tumor cells, is a significant indicator for predicting tumor immunogenicity [116,117,118]. Higher TMBs are significantly associated with better survival after anti-PD-1 therapy [118]. Typically, a high TMB indicates that tumor cells generate more neoantigens, which may correlate with a enhanced response to immune checkpoint inhibitor (ICI) therapy [119].

However, in tumors with a low TMB, AS-derived neoantigens provide a rich source of immunogenic targets, often more abundant than mutation-derived neoantigens [120,121,122,123,124,125,126]. A comprehensive study across 32 different cancer types identified approximately 1.7 neoepitope junctions (NJs) and approximately 0.6 single-nucleotide variant (SNV)-derived peptides per tumor sample predicted to bind to MHC I molecules, making them potential neoantigens [34]. The ability of AS-derived neoantigens to elicit immune responses highlights their potential as targets for immunotherapy, particularly in cancers with low TMB. Exploiting these diverse neoantigens generated through splicing dysregulation expands the scope of immune therapy. Recent reports have reported that RECTAS compounds can induce tumor cells to produce neoantigens with immune-activating properties [127,128]. These neoantigens include six specific splice isoform variants of the Kifc1, Nf1, Acbd4, Rfx7, Qpctl, and Nup153 proteins, which are produced due to aberrant AS, offering a promising method for innovative cancer immunotherapy strategies. In breast and ovarian cancers, more than 68% of cases express an AS-derived neoepitope, whereas only 30% of cases express a neoepitope derived from a SNV [34]. The high frequency of AS-derived neoantigens in low-TMB tumors, such as breast and ovarian cancers, underscores their potential as targets for immunotherapy, particularly when mutation-derived neoantigens are limited.

In addition to affecting neoantigen generation, AS also plays a role in modulating cytokine and cytokine receptor activity. For example, in asthma, the AS of IL-33 results in the generation of isoforms that drive inflammation. In human airway epithelial cells, AS of the IL-33 transcript with deletion of exons 3 and 4 (Δ exon 3,4) activates basophils and mast cells to drive type 2 inflammation in chronic stable asthma [129]. In most cases, however, isoforms of IL-2, IL-4, IL-6, and others might act as antagonists to their corresponding wild-type forms and block their activity [130]. Similarly, soluble isoforms of cytokine receptors, such as IL-6R and TNFR2, often function as inhibitors by blocking ligand signaling.

#### 3.10.2. AS in Immune Regulatory Molecules

AS plays a significant role in tumor immunity by generating tumor-specific neoantigens that can activate the immune system and, concurrently, enable tumor cells to evade immune surveillance. While AS-derived neoantigens have the potential to stimulate immune responses, some may lack immunogenicity, allowing tumors to evade immune detection [131,132,133]. Additionally, these neoantigens can trigger specific signaling pathways that reduce the expression of MHC I molecules on tumor cells’ surface, ultimately impeding the presentation of tumor antigens to T cells [134]. During the development of tumors, tumor cells often employ various mechanisms to reduce the expression of MHC I and decrease T cell recognition, facilitating immune evasion [135,136]. AS plays a crucial role in regulating immune checkpoint molecules, such as PD-1 and PD-L1, which are frequently dysregulated in tumors. In peripheral blood mononuclear cells (PBMCs) from healthy individuals, five splice isoforms of PD-1 mRNA have been identified [137,138], including PD-1∆ex2, PD-1∆ex3, PD-1∆ex2,3, PD-1∆ex2,3,4, and fIPD-1 [138,139]. In CRC, PD-L1 has distinct subtypes (e.g., PD-L1a, PD-L1b, PD-L1c) and soluble variants (e.g., PD-L1v229 and PD-L1v242) that act as decoys, Facilitating immune evasion by promoting PD-1/PD-L1 interactions [138,140,141,142]. Similarly, the AS of CTLA-4 generates soluble (sCTLA-4) and membrane-bound (mCTLA-4) forms [138,143,144], both of which contribute to immune escape.

SFs play a significant role in immune evasion mechanisms. For example, PTBP3 in SFs induces exon skipping in IL-18 in gallbladder cancer (GBC), producing a ΔIL-18 variant that reduces FBXO38-mediated PD-1 degradation in CD8^+^ T cells, thereby enhancing immune evasion [145]. This regulatory process substantially influences the diversity and specificity of immune-related gene expression, impacting the activity of cell surface receptors, including CD3, CD28, and CTLA-4, as well as kinases and phosphatases such as MAP4K2, MAP3K7 and CD45. Additionally, SFs regulate various transcription factors like GATA3 and FOXP3, and RBPs such as CELF2 and TIA-1 [28,146,147], essential for the proper development and response capabilities of the immune system.

Furthermore, tumor-derived splice isoforms can disrupt TCR signaling, hampering T cell activation and proliferation. Immune cells in the tumor microenvironment (TME), including regulatory T cells (Tregs) and tumor-associated macrophages (TAMs), are also influenced by tumor-specific AS events [148,149,150,151,152,153,154]. Notably, FOXP3, an important immunosuppressive molecule, exhibits two most abundant isoforms including full-length FOXP3 (FOXP3fl) and FOXP3 lacking exon 2 (FOXP3Δ2). FOXP3 lacking exons 2 and 7 (FOXP3Δ2Δ7) has been reported to inhibit other FOXP3 isoforms in a dominant negative manner, implying that exon 7 of FOXP3 is required for proper Treg cell function. The two different point mutations located near the intron 7 splice donor site result in the excision of FOXP3 exon 7 [155]. The splicing regulator USP39 modulates Treg function by maintaining CTLA-4 expression through lactate-mediated RNA splicing [156].

### 3.11. AS and Cancer Therapy

Advances in oncology research have shifted the focus of cancer treatment from traditional cytotoxic agents to targeted therapies. AS, a common event in cancer, has become a target for cancer therapy. By generating splice isoforms, AS contributes to tumorigenesis, immune evasion, and therapy resistance while also offering opportunities for innovative cancer treatments.

#### 3.11.1. Cancer Immunotherapy

ACT therapies, particularly CAR-T and TCR-T cell therapies, have shown significant potential in cancer treatment [157]. CAR-T cell therapy involves genetically engineered T cells that can recognize and attack neoantigens produced by AS, although challenges such as limited neoantigen recognition, T-cell persistence, and the immunosuppressive TME hinder their efficacy in solid tumors [158]. In contrast, TCR-T cells utilize TCRs to recognize TSAs presented by MHC molecules on the cell membrane or derived from within the cell [159]. This allows TCR-T cells to recognize a broader range of target antigens and induce more sustained immune synapse formation, making them particularly promising for solid tumors. For example, TCR-T cell therapy targeting the cancer-testis antigen NY-ESO-1 has shown encouraging results in various cancers, including NSCLC, CRC, hepatocellular carcinoma (HCC), and multiple myeloma [160,161,162]. Tumor-specific neoantigens generated by aberrant AS events presented by MHC I complexes provide a theoretical basis for TCR-T cell immunotherapy. Despite challenges such as neoantigen cross-reactivity, MHC restrictions, and mismatch risks, TCR-T cell therapy has demonstrated safety and efficacy, as evidenced by the FDA-approved kimtrak (tebentafusp-tebn) for the treatment of metastatic melanoma [163].

The lack of targetable antigens remains a significant challenge for immunotherapies such as CAR-T, TCR-T, and vaccines. While proteomics data often fail to detect AS-derived peptides due to NMD, advances in AI and computational biology now enable the screening of immunogenic shared neoantigens derived from dysregulated AS [164]. These neoantigens can serve as targets for CAR-T and TCR-T cell therapies, with engineered T cell administered to patients to increase immunotherapy efficacy. This approach holds great promise for overcoming current limitations and improving treatment outcomes. The integration of AS regulation with ICIs, CAR-T/TCR-T cell therapies, and personalized cancer vaccines represents a transformative strategy in cancer immunotherapy. By targeting immunogenic splice isoforms and neoantigens, these approaches offer new avenues for enhancing immune recognition and overcoming tumor immune evasion, paving the way for more effective and personalized cancer treatments.

**Figure 1:**
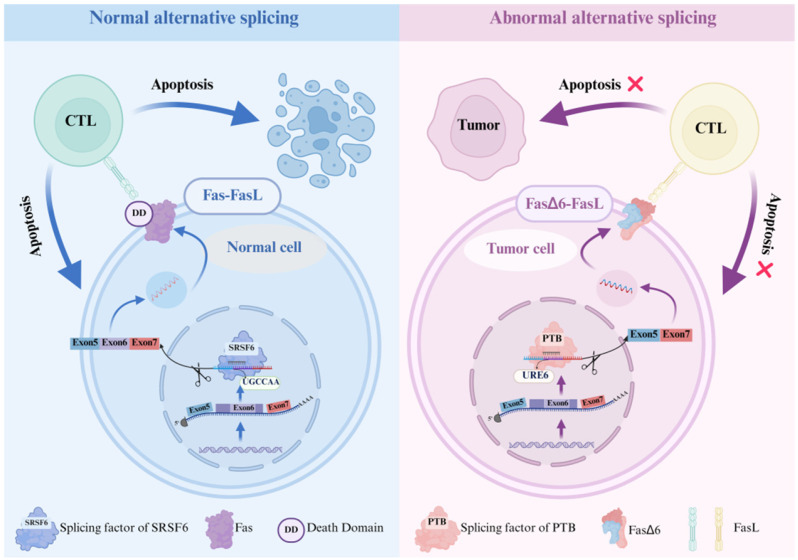
Normal and abnormal alternative splicing. Alternative splicing of the Fas gene was used as an example to compare normal and abnormal alternative splicing. Normal alternative splicing: The splicing factor SFSR6 binds to the UGCCAA region in exon 6 of the Fas gene, promoting the inclusion of exon 6. As a result, it is translated into a fully functional Fas protein, which promotes the apoptosis of tumor cells through the Fas-FasL pathway. Abnormal alternative splicing: The splicing factor PTB binds to the uridine-rich sequence located in exon 6 (URE6) of the CUCUCU region of the Fas gene, promoting the skipping of exon 6. Consequently, it is translated into the FASΔ6 protein. Moreover, this protein competes with the fully functional Fas protein for binding to FasL, thus facilitating the immune escape of tumors.

The regulation of AS plays a pivotal role in shaping the diversity and specificity of immune-related gene expression, influencing the activity of cell surface receptors (e.g., CD3, Fas, CTLA-4), kinases and phosphatases (e.g., MAP4K2, MAP3K7, CD45), and transcription factors (e.g., GATA3 and FOXP3) as well as RBPs (e.g., CELF2 and TIA-1) (Table 2). Numerous studies have shown that during the process of cell apoptosis, Fas is regulated by several SFs (e.g., SRSF6, hnRNPC, and PTB). In normal AS, SFSR6 binds to the UGCCAA region in exon 6 of the Fas gene [165]. This binding promotes the inclusion of exon 6. A fully functional Fas protein is subsequently translated. This Fas protein promotes the apoptosis of normal cells through the Fas/FasL pathway. In contrast, in abnormal AS, PTB binds to the uridine-rich sequence (URE6) of the CUCUCU region located in exon 6 of the Fas gene [166]. This binding promotes the skipping of exon 6. As a result, the FASΔ6 protein is translated. Additionally, this protein competitively binds to FasL with the fully functional Fas protein, thereby facilitating the immune escape of tumors (Figure 1).

The combined application of neoantigens produced by aberrant AS and ICIs represents an emerging therapeutic strategy [184,185]. This strategy is based on the immunogenic neoantigens generated by tumor-specific AS, which can be recognized by the immune system as “nonself” components, thereby stimulating an antitumor immune response [186,187]. For example, studies have shown that RBM39 of the SF degrader Indisulam can induce tumor cells to produce neoantigens that are presented by MHC I to stimulate an antitumor immune response. The combination of Indisulam and PD-1 antibodies has been shown to synergistically inhibit tumor growth, highlighting the therapeutic potential of integrating AS regulation with ICIs.

#### 3.11.2. SSOs and Cancer Therapy

SSOs are small, synthetic nucleic acids that interfere with the pre-mRNA splicing process by disrupting RNA-RNA or protein-RNA interactions essential for the splicing machinery [32]. SSOs differ from siRNAs in regulating gene expression through splicing control rather than RNA degradation. AS has emerged as a promising target for cancer therapy, with SSOs being able to modulate the expression of critical oncogenic isoforms. By redirecting splicing toward proapoptotic isoforms, SSOs have demonstrated significant antitumor effects in both cell culture and xenograft models. For example, targeting Bcl-x, HER4, MDM4, and STAT3 splicing variants with SSOs has shown significant antitumor effects in cell culture and xenograft models. STAT3 exists in two main isoforms, namely, full-length STAT3α and truncated STAT3β, which are generated by the AS of exon 23 [35]. STAT3β acts as a dominant-negative regulator of transcription and promotes apoptosis. SSOs that redirect splicing from STAT3α to STAT3β have been shown to increase cancer cell death and induce tumor regression in xenograft models. In contrast to the effect mediated by total STAT3 knockdown-induced FSD-NMD, SSOs displayed a unique STAT3β signature, with the downregulation of specific targets. The Bcl-x gene produces two primary protein isoforms with opposing functions: the antiapoptotic protein Bcl-xL and the proapoptotic protein Bcl-xS. Bcl-xL is elevated in various cancers and contributes to resistance to a wide spectrum of chemotherapeutic agents. Conversely, Bcl-xS counteracts the antiapoptotic effects of Bcl-xL. SSOs that shift Bcl-x splicing from Bcl-xL to Bcl-xS induce apoptosis and increase sensitivity to chemotherapy in cancer cells cultured in vitro and inhibit tumor growth in vivo [188]. The insulin receptor (IR) is differentially spliced at exon 11 to generate two distinct isoforms—namely, IR-B (which includes exon 11) and IR-A (which excludes exon 11). SSOs that target IR splicing, either by modulating regulatory elements such as CELF1 or directly restoring splicing from IR-A to IR-B, have been shown to inhibit osteosarcoma growth and resistance to anoikis [189,190].

#### 3.11.3. Targeting Novel Splice Variants

Given the importance of mRNA AS and the fundamental role of the spliceosome posttranscriptionally, the spliceosome has attracted attention as an anticancer target. Small-molecule compounds such as pladienolides [16] modulate the SF3b subunit of the spliceosome, interfering with its function in a dose-dependent manner. Drugs such as Indisulam (E7070), a small-molecule inhibitor, degrade RBM39, leading to widespread aberrant splicing and translation of key downstream effector proteins [191]. Indisulam has shown therapeutic effects in various cancer types, such as T-cell acute lymphoblastic leukemia (T-ALL) [109], high-grade serous ovarian cancer (HGSC) [110], and head and neck squamous cell carcinoma (HNSCC) [111]. Tepotinib [192] is a highly selective MET tyrosine kinase inhibitor, targeting the specific oncogenic driving mechanism of MET gene exon 14 skipping mutations in NSCLC [42]. This mutation leads to the deletion of exon 14 through AS of RNA, causing the MET receptor tyrosine kinase to avoid degradation and remain continuously activated, thereby abnormally activating the downstream pro-proliferative signaling pathways. As the first targeted drug approved for this target, Tepotinib precisely inhibits the kinase domain of the MET protein, blocking its abnormal signal transduction and demonstrating significant antitumor activity. This breakthrough validates the druggability of abnormal AS as a target for cancer treatment, marking a new stage in the clinical application of targeted therapies developed based on the RNA splicing mechanism.

Aberrant splicing in tumor cells generates neoantigens that can be presented by MHC I molecules, enhancing T cell recognition and attack [193]. Modulating AS with drugs such as Indisulam increases the presentation of these neoantigens, stimulating antitumor immune responses. Preclinical studies have demonstrated that combining Indisulam with PD-1 antibodies synergistically inhibits tumor growth, highlighting the potential of integrating AS regulation with ICIs for improved cancer immunotherapy [194].

## 4. Innovative Technologies in the Study of AS

Tumor-associated aberrant splicing events contribute to approximately 15–30% of oncogenic mutations, with frameshift proteins derived from these events potentially activating the immune system through neoantigen presentation [195]. Traditional genomic methods, such as whole-exome sequencing (WES), detect only approximately 1.5% of tumor neoantigens, whereas splicing-driven neoantigens account for 34–83% of cases [196]. Recent advances in computational biology and multi-omics technologies have shifted research focus toward high-precision splicing analysis tools and cross-omics integration strategies to systematically decode tumor-specific splicing events and their immunotherapeutic potential.

### 4.1. Splicing Analysis Tools and Databases

The diversification of differential splicing detection tools has propelled cancer research. Replicate Multivariate Analysis of Transcript Splicing (rMATS) [197] employs likelihood ratio tests and ΔPSI quantification to identify five classical splicing events, with outputs (e.g., BRAF exon skipping in melanoma) serving as starting points for neoantigen screening.

Whippet [198], leveraging lightweight algorithms, enables efficient quantification of complex splicing events on standard laptops, revealing that high-entropy AS events affect up to 40% of human genes.

Psichomics [199] integrates multi-omics data (RNA-seq, clinical outcomes) from TCGA, supporting AS event detection (e.g., exon skipping/intron retention), isoform quantification (via PSI values), and survival analysis (Cox models, KM curves). Its tumor-specific splicing outputs (e.g., exon-skipped aberrant transcripts) encode neoantigens, offering candidates for immunotherapy.

AS Cancer Atlas (https://ngdc.cncb.ac.cn/ascancer, accessed on 25 March 2025) [200], a core repository, catalogs 17,842 experimentally validated cancer-associated AS events, including splice sites, isoform expression, and prognostic data. This helps researchers gain deeper insights into splicing regulation and discover potential therapeutic targets.

### 4.2. Neoantigen Prediction Innovations

Splicing-driven neoantigen discovery relies on algorithmic advancements and multi-dimensional validation. The Spliced Neo Antigen Finder (SNAF) [201] tool integrates DeepImmuno-CNN and BayesTS models. Notably, more than 90% of more than 500 melanoma patients presented with shared splice neoantigens.

Researchers from the Children’s Hospital of Philadelphia (CHOP) and the University of California, Los Angeles (UCLA), have collaboratively developed a computational platform called isoform peptides from RNA splicing for immunotherapy target screening (IRIS) [202,203], that the platform combines tumor/normal differential expression (fold change >5) and HLA-I affinity prediction, prioritizing 48 TCR targets in neuroendocrine prostate cancer (NEPC) from 2939 splicing events.

A research team from the University of Lausanne has employed a revolutionary machine learning (ML) [204] method to significantly enhance the ability to identify immunogenic neoantigens and mutations, which is crucial for the development of personalized cancer immunotherapies. This technology has reprocessed WES and RNA-seq data from 112 cancer patients from the National Cancer Institute (NCI), 8 patients from the Tumor Neoantigen Selection Alliance (TESLA), and an internal dataset of 11 patients. The results revealed over 46,000 somatic single-nucleotide mutations and approximately 1.78 million new peptides, of which 212 mutations and 178 peptides showed immunogenicity. Moreover, the classifier trained on the large NCI dataset was able to accurately predict the immunogenicity of neoantigens across multiple datasets, and this method outperformed previous approaches in terms of orthogonal features, increasing the accuracy of predictions and the number of high-ranking immunogenic peptides by up to 30%. For specific details, refer to Table 3.

### 4.3. Multi-Omics Integration Strategy

Initial screening is conducted using NGS-based WES and RNA-seq (Illumina NovaSeq X Plus) to identify high-frequency mutations and differentially expressed genes [205,206,207,208,209,210,211]. Candidate splicing events are pinpointed via rMATS [197] and Whippet [198]. PacBio SMRT (10–15 kb reads) resolves complex structural variants (e.g., full-length ALK fusion isoforms), while HiFi sequencing [212] (CCS mode, 10–25 kb reads, accuracy ≥99.9%) validates low-abundance splice variants. Cross-referencing with AS Cancer Atlas identifies tumor-specific frameshift transcripts. Orbitrap Astral mass spectrometry (MS) [213,214,215,216] (DIA mode, 0.1 attomolar sensitivity) detects frameshift-derived peptides, followed by Spectronaut 16.0 spectral matching and XGBoost 3.0.1 for HLA-I/II binding prediction. Final immunogenicity validation employs the IRIS [202,203] platform and in vitro T-cell activation assays, establishing a closed-loop system from splicing discovery to therapeutic targets. This strategy increases neoantigen detection rates compared to traditional methods.

From differential splicing tools (rMATS, Whippet) to neoantigen algorithms (SNAF, XGBoost), and from long-read sequencing (HiFi) to multi-omics integration strategy, technological advancements are driving clinical translation. Future breakthroughs in cost-effective long-read sequencing, single-cell proteomics, and causal inference models will further overcome bottlenecks, enabling scalable personalized immunotherapy based on splicing-derived neoantigens.

## 5. Challenges and Difficulties

Despite the promising use of neoantigens in cancer immunotherapy, their discovery and clinical application remain limited due to several challenges. Tumor heterogeneity and dynamic changes lead to inconsistent neoantigen expression, complicating their identification and use as therapeutic targets. Additionally, only a subset of neoantigens possesses sufficient immunogenicity to trigger effective immune responses, whereas tumor immune escape mechanisms further reduce immune recognition. Predicting neoantigens requires advanced computational biology methods, and experimental validation is both time-consuming and costly. Other hurdles include individual variations in immune responses; technical limitations in high-throughput data analysis; and the complexities of clinical translation, such as production, safety, efficacy, and regulatory approval. These factors collectively create a multistep, interdisciplinary process that demands continuous technological innovation and cross-disciplinary collaboration.

## 6. Conclusions and Prospects

Aberrant AS in tumor cells plays a dual role in cancer immunotherapy. On the one hand, it can facilitate immune evasion by enabling tumor cells to escape immune surveillance. On the other hand, it generates immunogenic neoantigens that are presented by MHC I molecules to T cells, triggering antitumor immune responses. To expand the repertoire of potential immunotherapeutic antigens, recent studies have explored aberrant splicing events across multiple cancers as an additional source of TSAs. These cancer-specific splicing events, otherwise known as NJs, are prevalent in cancer cells and capable of generating novel TSAs that potentiate CD8^+^ T cell-mediated expansion and responses in select cancer types. When combined with ICIs (e.g., PD-1 or PD-L1 inhibitors), these neoantigens have synergistic potential in inhibiting tumor growth, laying the groundwork for personalized immunotherapy. However, neoantigens may lack sufficient immunogenicity, or the quantity may not be enough to activate an effective antitumor immune response. In addition, tumor cells may also regulate splice isoforms to evade immune surveillance. Although TCR-T and CAR-T cell therapies have shown promise in immunotherapy targeting neoantigens, they also face challenges such as MHC restrictions, potential mismatch risks, and immune suppression of the TME.

Advancements in computational tools and platforms, such as SNAF, ML, and IRIS, are revolutionizing the identification of immunogenic neoantigens. These tools enhance the precision of immunotherapy and improve patient outcomes. Additionally, AI algorithms and high-throughput sequencing technologies are enabling the design of personalized neoantigen vaccines tailored to the unique splicing profiles of individual tumors. Clinical trials have demonstrated that these vaccines can stimulate potent immune responses, particularly when combined with ICIs.

Despite challenges such as limited neoantigen immunogenicity, immune suppression mediated by the TME, and the high costs and technical demands of personalized therapies, ongoing clinical trials and technological innovations are paving the way for more effective and precise treatments. The discovery and utilization of neoantigens, along with strategies to modulate AS, will be pivotal in advancing cancer immunotherapy. As our understanding of tumor-specific splicing events deepens, more therapies targeting immunogenic neoantigens are expected to emerge. These neoantigens not only provide targets for personalized vaccines but also increase the efficacy of ICIs and CAR-T and TCR-T cell therapies. The development of personalized neoantigen vaccines represents a significant step toward precision medicine.

## Figures and Tables

**Table 1:** Some of abnormal alternative splicing in various malignant tumors.

Cancer	Target Gene	AS Isoform	Exon/Intron	AS Type	Function	Reference
Breast Cancer	*ER*	ERα36	exon 7, 8	Exon skipping, Inclusion	Resistance to endocrine therapies.	[90,91,92]
		ERα46	exon 1, exon 9			
		ERΔ7	exon 7			
	*HER2*	Δ16HER2	exon 20	Exon skipping	Increased transforming ability.	[69]
	*CD44*	CD44v2-v10	exon v2-v10	Exon Inclusion	BC growth and metastasis.	[72,93]
		CD44v3-v10	exon v3-v10			
		CD44v8-v10	exon v8-v10			
		CD44v6	exon v6			
Colorectal Cancer	*KRAS*	KRAS4A	exon 4A	Exon Inclusion	Cancer stemness.	[94]
		KRAS4B	exon 4A	Exon skipping	Response to endoplasmic reticulum stress.	
	*KLF6*	KLF6-SV2	exon 2	Exon skipping	CRC cell proliferation and apoptosis.	[95]
Lung Cancer	*RBM4*	RBM4-S	exon3	Exon skipping	Activation of the SRSF1-mTORC1 pathway promotes NSCLC cell growth.	[2]
	*MET*	METΔex14	exon 14	Exon skipping	Oncogenic activity.	[96]
	*HER2*	HER2D16	exon 16	Exon skipping	A mediator of osimertinib resistance in patients with metastatic EGFR-mutant NSCLC.	[97]
	*TP53*	P53β	exon 9β	Exon Inclusion	Cellular senescence.	[98,99,100]
		p53γ	exon 9γ		Cell differentiation/Antioxidant response.	
		p53Ψ	intron 6	Alternative 3′ splicing	Epigenetic regulation.	
		Δ40p53	intron 2	Intron Retention	Apoptosis.	
		Δ133p53	intron 4		A novel transcriptional enhancer of T-cell effector function.	
		Δ160p53	intron 4		Tumor cell migration and invasion.	
	*Bcl—X*	Bcl-Xs	exon 2	Alternative 5′ splicing	Resistance against chemotherapeutic agents.	[101]
Hematological Malignancies	*BCL—2*	BCL-2β	exon 3	Exon skipping	Antiapoptotic.	[86]
Ovarian Cancer	*BCL—2*	BCL2L12-L	exon 3	Exon Inclusion	Apoptosis.	[102]
		BCL2L12-S	exon 3	Exon skipping		
Hepatocellular Carcinoma	*KLF6*	KLF6-SV1	exon 2	Alternative 5′ splicing	Cancer metastasis, progression.	[103]
	*CDC25A*	CDC25A ΔE6	exon 6	Exon skipping	HCC growth.	[104]
	*ADRM1*	ADRM1-ΔEx9	exon 9	Exon skipping	Ubiquitin proteasome specificity.	[105]

**Table 2:** Splicing factors that regulate genes related to immune cells.

Splicing Factors	Types of Immune Cells	Affected Genes	References
SRSF1, SFPQ, CELF2	T cell	Irf7, Il27ra, CD45	[167,168,169]
PTB, HuR, hnRNPC, RBM5	T cell	Fas, PD-L1, CTLA-4	[170,171,172,173,174]
DDX39B	T cell	FOXP3	[175]
hnRNPL	B cell	MYC, E2F	[176]
SF3B1	B cell	BCL2, MYC	[177,178]
TCF3	B cell	E12, E47	[179,180]
KIR	NK cell	___	[181]
PTBP1	DCs	MHC II	[182,183]

**Table 3:** Some of the innovative technologies for detecting aberrant alternative splicing events, neoantigen immunogenicity, and neoantigen target screening.

Technology/Database	Core Functions/ Technical Features	Advantages	Limitation	Complementary Technologies	Clinical Translational Value	References
rMATS	Quantifies 5 classical splicing events using ΔPSI	High sensitivity, standardized pipeline	Requires replicate samples, cannot resolve complex isoforms	Whippet	Initial screening of splicing events linked to high-frequency mutations	[197]
Whippet	Lightweight algorithm for high-entropy AS detection	Rapid single-sample analysis, covers 40% human genes	Low sensitivity for weakly expressed genes, lacks clinical data integration	PacBio SMRT	Large-scale screening of potential therapeutic targets	[198]
Psichomics	TCGA integration with Cox survival modeling	Direct patient prognosis association, target prioritization	Public database dependency, low flexibility	AS Cancer Atlas	Prognostic biomarker discovery	[199]
AS Cancer Atlas	Pan-cancer AS event database integrating TCGA/GTEx (33 cancer types) with survival-mutation links	Interactive visualization of splicing-clinical correlations	Infrequent updates, sparse data for rare cancers	Psichomics	Identification of pan-cancer splicing targets and therapy-response biomarkers	[200]
SNAF	DeepImmuno-CNN + BayesTS framework for splicing-derived neoantigens	High specificity (>85%), high shared antigen ratio (>90%)	RNA-seq coverage dependency, lacks MS validation	IRIS	Development of universal TCR therapies	[201]
IRIS	Differential expression + HLA-I affinity filtering	30% reduced false positives, high verifiability	Requires matched normal samples, HLA typing constraints	ML	Personalized TCR therapy development	[202,203]
ML	XGBoost + immunogenicity classifier	30% AUC improvement, strong generalizability	Data-hungry, computationally intensive	Orbitrap Astral MS	Enhanced personalized vaccine design	[204]
NGS	Short-read detection of high-frequency mutations/Differential genes	Cost-effective, standardized workflow	Misses splicing drivers	rMATS/Whippet	Foundational mutation profiling	[205,206,207,208,209,210,211]
PacBio SMRT	Long-read (10–15 kb) resolution of complex SVs	Error-free assembly, full-length isoform detection	High cost, low throughput	PacBio HiFi	Guidance for fusion protein targeting	[212]
PacBio HiFi	High-fidelity long-reads (≥99.9%) for rare isoform validation	0.1 attomolar sensitivity	Large storage requirements	Orbitrap Astral MS	Enhanced neoantigen authenticity validation	[212]
Orbitrap Astral MS	DIA-MS detection of frameshift peptides	Antibody-free direct validation	Database dependency, low-abundance peptide challenges	Spectronaut	Confirmation of neoantigen presentation	[213,214,215,216]
SUPPA2	AS analysis via transcript abundance (PSI calculation)	Single-sample compatibility, no replicates required	Limited by transcript reconstruction accuracy, low sensitivity for rare isoforms	Salmon	Rapid identification of prognosis-associated splicing events	[217]
LeafCutter	Reference-free splicing analysis using intron excision sites	Novel isoform discovery without exon annotation	High sequencing depth requirement, limited complex SV resolution	STAR	Detection of non-canonical splicing drivers	[218]
pVACtools	NetMHCpan + expression filtering + immunogenicity scoring	Open-source multi-threading support	Manual parameter tuning, lacks long-read integration	NeoPredPipe	Personalized vaccine candidate prioritization	[219]
MHCflurry2.0	DL-based HLA-I/II affinity prediction	Covers >10,000 HLA alleles, cross-validation support	Reduced accuracy for rare HLA types	PrimeRank	Improved T-cell response prediction	[220]

## Data Availability

Not applicable, no new data are presented in this review.

## Abbreviations

ACT	Adoptive Cell Therapy.
AML	Acute Myeloid Leukemia.
AS	Alternative Splicing.
AS Cancer Atlas	Alternative Splicing Cancer Atlas.
BC	Breast Cancer.
BCR	B-cell Receptor.
CAR-T	Chimeric Antigen Receptor T-cell.
CCS	Circular Consensus Sequencing.
CRC	Colorectal Cancer.
CHOP	Children’s Hospital of Philadelphia.
EMT	Epithelial-to-Mesenchymal Transition.
ESEs	Exonic Splicing Enhancers.
ESSs	Exonic Splicing Silencers.
ERs	Estrogen Receptors.
ER	Endoplasmic Reticulum.
FDSIs	Functionally Distinct Splice Isoforms.
FLT3-ITDs	FLT3 Internal Tandem Duplications.
GBM	Glioblastoma.
GBC	Gallbladder Cancer.
HCC	Hepatocellular Carcinoma.
HGSC	High-grade serous ovarian cancer.
HNSCC	Head and neck squamous cell carcinoma.
hnRNPs	Heterogeneous Nuclear Ribonucleoproteins.
ISEs	Intronic Splicing Enhancers.
ISSs	Intronic Splicing Silencers.
ICB	Immune Checkpoint Blockade.
ICI	Immune Checkpoint Inhibitor.
Ig	Immunoglobulin.
IRIS	Isoform Peptides from RNA Splicing for Immunotherapy Target Screening.
ISR	Integrated Stress Response.
IR	Insulin Receptor.
LUAD	Lung Adenocarcinoma.
ML	Machine Learning.
MHC	Major Histocompatibility Complex.
m6A	N6-methyladenosine.
MDS	Myelodysplastic syndromes.
MS	Mass Spectrometry.
NJs	neojunctions.
NMD	Nonsense—mediated Decay.
NSCLC	Non-small cell lung cancer.
NGS	Next-Generation Sequencing.
NEPC	Neuroendocrine prostate cancer.
NCI	National Cancer Institute.
ORFs	Open Reading Frames.
PBMCs	Peripheral Blood Mononuclear Cells.
PDAC	Pancreatic ductal adenocarcinoma.
PTCs	Premature Termination Codons.
RBPs	RNA-binding proteins.
RBM	RNA-binding Motif RNA.
ROS	Reactive Oxygen Species.
rMATS	replicate Multivariate Analysis of Transcript Splicing.
snRNAs	small nuclear RNAs.
snRNPs	small nuclear ribonucleoproteins.
SAVs	Splice-altering Variants.
SNAF	Spliced Neo Antigen Finder.
SNV	Single-Nucleotide Variant.
SMRT	Single Molecule, Real-Time.
SRs	Serine/Arginine-rich Proteins.
SSOs	Splice-switching Oligonucleotides.
TAMs	Tumor Associated Macrophages.
TCR	T-cell Receptor.
TCR-T	T-cell Receptor Engineered T-cell.
Tregs	Regulatory T Cells.
TME	Tumor Microenvironment.
TMB	Tumor Mutational Burden.
TKIs	Tyrosine Kinase Inhibitors.
TSAs	Tumor-Specific Antigens.
T-ALL	T-cell acute lymphoblastic leukemia.
TESLA	Tumor Neoantigen Selection Alliance.
URE6	Uridine-rich sequence located in exon 6.
UCLA	University of California, Los Angeles.
WES	Whole-exome sequencing.
